# Allogeneic hematopoietic stem cell transplantation is associated with improved long-term survival and molecular MRD clearance in *KMT2A*-altered AML

**DOI:** 10.1080/07853890.2026.2758994

**Published:** 2026-11-12

**Authors:** Yi Chen, Zhiwei Ding, Shuxia Zhang, Xiaofan Li, Ruyu Cai, Danhui Fu, Fenge Yang, Shaoyuan Wang, Yong Wu, Nainong Li, Jing Zheng

**Affiliations:** Fujian Provincial Key Laboratory on Hematology, Fujian Institute of Hematology, Fujian Medical University Union Hospital, Fuzhou, China

**Keywords:** Acute myeloid leukemia, *KMT2A* alterations, allogeneic hematopoietic stem cell transplantation, minimal residual disease, prognosis

## Abstract

**Background:**

*KMT2A*-altered acute myeloid leukemia (AML) is a rare, aggressive high-risk subtype. The prognostic value of flow-cytometric phenotypic MRD versus *KMT2A* gene-specific molecular MRD, and the role of allogeneic hematopoietic stem cell transplantation (Allo-HSCT), remain poorly defined.

**Methods:**

This single-center retrospective cohort enrolled 155 newly diagnosed adolescent and adult patients with *KMT2A*-altered AML. Treatment response, MRD status, and survival were analyzed by Kaplan–Meier and multivariate Cox regression; time-dependent Cox regression was used as a sensitivity analysis to account for guarantee-time bias.

**Results:**

Among 153 evaluable patients, the complete remission rate was 70.7% and overall response rate 78.9%, with median RFS of 12.4 months and OS of 16.4 months. After excluding transplanted patients, the three non-transplant regimens yielded similar survival, indicating no inherent advantage of induction regimens. Flow-MRD-negative patients had superior OS, and concurrent *KMT2A* molecular MRD negativity conferred the best outcomes. Allo-HSCT was associated with a markedly higher rate of sustained molecular MRD negativity (74.6% vs. 14.4%, *p* < 0.0001). On multivariate analysis, persistent molecular MRD positivity and absence of Allo-HSCT independently predicted inferior RFS and OS; after time-dependent adjustment, Allo-HSCT showed only a non-significant trend, while molecular MRD positivity remained the dominant adverse prognostic factor.

**Conclusion:**

In *KMT2A*-altered AML, sustained *KMT2A* molecular MRD negativity is the key determinant of favorable long-term survival. Allo-HSCT promotes molecular MRD clearance and may serve as a consolidation strategy to achieve deep molecular remission.

## 1. Introduction

Acute myeloid leukemia (AML) is a genetically heterogeneous hematological malignancy, whose clinical prognosis and therapeutic response are profoundly determined by molecular cytogenetic abnormalities [1,2]. Gene alterations in *KMT2A*, including gene rearrangements (*KMT2Ar*) and partial tandem duplication (*KMT2A-PTD*), represent a classic high-risk genetic subtype in adult AML [3–5]. Although *KMT2Ar* and *KMT2A-PTD* are distinct biological entities with divergent underlying molecular mechanisms, both confer high-risk disease features in adult AML and share an unfavorable prognosis in conventional chemotherapy cohorts; therefore, we pooled these two subtypes in the present analysis. Patients diagnosed with *KMT2A*-altered AML typically present with elevated tumor burden, suboptimal responses to conventional chemotherapy, and a high cumulative risk of disease relapse, leading to unsatisfactory long-term survival outcomes [6,7]. Despite substantial progress in molecular classification and targeted therapeutic exploration in AML, unified prognostic stratification criteria and optimized treatment decision-making strategies for *KMT2A*-altered AML remain insufficient, largely due to the lack of real-world cohort evidence with adequate sample size. Emerging menin-*MLL* targeted inhibitors represent promising novel therapies for *KMT2A*-driven AML; the interplay between molecular MRD status, allogeneic hematopoietic stem cell transplantation, and these modern targeted pathways warrants further investigation.

Minimal residual disease (MRD) has been universally validated as the most reliable dynamic prognostic biomarker for evaluating treatment response and predicting relapse risk in AML [8,9]. Traditional morphological bone marrow assessment can only reflect macroscopic hematological remission and lacks sufficient sensitivity to detect trace residual leukemic cells. In contrast, molecular MRD detection enables precise evaluation of deep molecular remission, which complements morphological assessment and refines prognostic stratification [10–13]. Nevertheless, the independent prognostic value of *KMT2A* molecular MRD status has not been fully verified in adult AML populations. Moreover, the efficacy discrepancies among intensive chemotherapy, venetoclax-combined hypomethylating agent regimens, and low-intensity therapies, as well as the consolidative effect of allogeneic hematopoietic stem cell transplantation (Allo-HSCT), require systematic comparative analysis in patients with diverse *KMT2A* aberrations.

This retrospective single-center cohort study included 155 newly diagnosed adult patients with *KMT2A*-altered AML. The present study aimed to compare the clinical efficacy and survival outcomes of different frontline therapeutic regimens, and to screen independent clinical and molecular prognostic factors affecting RFS and OS. The findings of this study provide real-world clinical evidence for MRD-guided prognostic stratification and individualized transplantation-based curative strategies for high-risk *KMT2A*-altered AML.

## 2. Methods

### 2.1. Study population

This retrospective study enrolled adolescent and adult patients with newly diagnosed *KMT2A*-altered AML who received treatment at Fujian Medical University Union Hospital from January 2016 to December 2025. Clinical, laboratory, genetic, and follow-up data were systematically collected and reviewed. All procedures were performed in accordance with the principles outlined in the Declaration of Helsinki. The study was approved by the Ethics Committee of Fujian Medical University Union Hospital (protocol number: 2026KY803), which waived the requirement for written informed consent because no study-related interventions were performed and all patient data were fully de-identified before analysis.

### 2.2. Definition of AML and genetic mutations

AML diagnosis was established according to the 2016 revised World Health Organization (WHO) classification criteria for myeloid neoplasms and acute leukemia [14]. Secondary AML (s-AML) was defined as AML evolved from prior myeloproliferative neoplasms, while therapy-related AML (t-AML) referred to AML occurring after previous chemotherapy or radiotherapy for other malignancies. Patients with blast-phase chronic myeloid leukemia were excluded. A complex karyotype was defined as the presence of at least three unrelated chromosomal abnormalities.

Genetic profiling was performed using a custom myeloid next-generation sequencing (NGS) panel (Kangsheng Global Medical Technology, Beijing, China) on an Illumina NextSeq 550 platform to identify *KMT2A* rearrangements and other somatic mutation. Quantification of *KMT2A* fusion transcripts and *KMT2A-PTD* was conducted *via* reverse transcription quantitative PCR (RT-qPCR) on an Applied Biosystems^™^ 7500 Real-Time PCR System, with a lower limit of detection of 10^−4^. Detailed RT-qPCR experimental procedures, reagents and instrument information are provided in Supplementary Methods.

### 2.3. Treatment protocols

Three frontline therapeutic strategies were applied in this cohort. The intensive chemotherapy (IC) regimen followed a standard 3 + 7 protocol consisting of idarubicin or daunorubicin combined with cytarabine. Patients who achieved complete remission (CR) or complete remission with incomplete hematologic recovery (CRi) received intermediate- or high-dose cytarabine as consolidation therapy. The hypomethylating agent plus venetoclax (VA) regimen comprised azacitidine (75 mg/m^2^ daily for 7 consecutive days) combined with venetoclax. Venetoclax was administered at 100 mg on day 1, escalated to 200 mg on day 2, and maintained at 400 mg daily from day 3 to day 28. For patients achieving CR, dual-agent combination treatment was continued until disease progression or intolerable toxicity. Low-intensity regimens included single-agent hypomethylating agents (azacitidine or decitabine) or hypomethylating agents combined with low-dose chemotherapy, which were continuously administered for consolidation in responding patients. For patients harboring *FLT3* mutations, targeted inhibitors (sorafenib or gilteritinib) were incorporated into frontline regimens. Patients who failed initial induction therapies received second-line treatment. Detailed initial treatment information is summarized in Supplementary Table 1. In this cohort, some patients received Allo-HSCT. The key requirements for transplantation included the availability of a suitable donor, adequate organ function, acceptable ECOG performance status, disease status suitable for transplantation, and patients’ voluntary informed consent to undergo Allo-HSCT.

### 2.4. Definition of response and outcomes

Treatment response was evaluated based on the 2022 European LeukemiaNet (ELN) response criteria for AML [15]. Peripheral blood routine examinations were performed per treatment cycle, and bone marrow aspiration was conducted every one to two cycles to assess treatment response. The overall response rate (ORR) was defined as the composite rate of CR, CRi, and partial remission (PR). Multiparametric flow cytometry (BD FACSCanto, BD Biosciences, USA) was applied for phenotypic MRD detection with a sensitivity of 10^−4^. Molecular MRD was determined by quantitative detection of *KMT2A* fusion transcripts. Disease relapse was defined as the reappearance of leukemic blasts exceeding 5% in bone marrow or peripheral blood after CR/CRi achievement, or the emergence of extramedullary leukemia lesions. The follow-up cut-off date for this study was July 17, 2026. Data for patients lost to follow-up were censored at the last documented contact. Relapse-free survival (RFS) was calculated from the date of CR/CRi achievement to the date of relapse, disease progression, or last follow-up. Overall survival (OS) was defined as the interval from initial diagnosis to death from any cause or the final follow-up.

### 2.5. Statistical analysis

Categorical variables were compared using the chi-square test. Continuous variables with normal distribution were analyzed using the Student’s *t*-test, while non-normally distributed data were evaluated using nonparametric tests. Restricted cubic spline (RCS) Cox models were applied to assess potential non-linear associations between continuous biomarkers (age, lactate dehydrogenase, peripheral white blood cell count, bone marrow blast percentage) and survival endpoints. Three knots were used for each spline term. The predefined age cutoff of 60 years was retained for the primary prognostic analysis. Survival curves were generated *via* the Kaplan–Meier method and compared using the log-rank test. Cox proportional hazards regression models were utilized to screen univariate and multivariate prognostic factors for RFS and OS. The data analysis for this retrospective study was initiated on July 30, 2026. All statistical analyses and graphical visualizations were performed using SPSS 21.0, GraphPad Prism 9.5, and R software (version 4.6.1). A two-tailed *p* value < 0.05 was considered statistically significant.

## 3. Results

### 3.1. Baseline patient characteristics

A total of 155 adult patients with newly diagnosed *KMT2A*-altered AML were enrolled in the present study, with a median age of 48 years (range: 14–79 years) and a male-to-female ratio of 78:77. Among the overall cohort, 135 patients (87.1%) had de novo AML, 8 (5.2%) had secondary AML (s-AML), and 12 (7.7%) had therapy-related AML (t-AML). At initial diagnosis, 66 patients (42.6%) presented with infectious symptoms, 57 (36.8%) presented with anemia, and 20 (12.9%) presented with bleeding manifestations. Baseline laboratory examinations revealed a median peripheral white blood cell (WBC) count of 13.0 (0.2–239.1) × 10^9^/L, a median hemoglobin level of 73 (25–161) g/L, and a median platelet count of 45 (5–399) × 10^9^/L. The median serum lactate dehydrogenase (LDH) level was 392 (52–10234) IU/L, and the median bone marrow blast percentage was 73.5% (16.5%–98.5%). Coagulation abnormalities were prevalent across the cohort, including prolonged prothrombin time in 83 patients (53.5%), prolonged activated partial thromboplastin time in 86 patients (55.5%), and decreased fibrinogen levels in 16 patients (10.3%). Regarding *KMT2A* genetic aberrations, *KMT2A-PTD* was identified in 65 patients (41.9%), followed by *KMT2A::MLLT3* (38 cases, 24.5%), *KMT2A::MLLT4* (23 cases, 14.8%), *KMT2A::ELL* (18 cases, 11.6%), *KMT2A::MLLT10* (9 cases, 5.8%), and *KMT2A::MLLT6* (2 cases, 1.3%). The most frequently co-occurring mutations included *FLT3-ITD* (31 cases, 20.0%), *NRAS* (22 cases, 14.2%), *ASXL1* (20 cases, 12.9%), *DNMT3A* (20 cases, 12.9%), and *KRAS* (17 cases, 11.0%). Mutations in *IDH2*, *RUNX1*, *TET2*, and *STAG2* were detected in 14 patients each (9.0%). *NPM1* mutation was rare, occurring in only 3 young patients (1.9%). Chromosome +8 abnormality was detected in 9 patients (5.8%), and a complex karyotype was observed in 8 patients (5.2%). According to the 2022 ELN risk stratification system, 30 patients (19.4%) were classified as intermediate-risk and 125 patients (80.6%) as adverse-risk. Detailed baseline clinical and genetic features are summarized in Table 1 and Figure 1. The patient screening and enrollment flowchart is shown in Figure 2.

**Figure 1.**
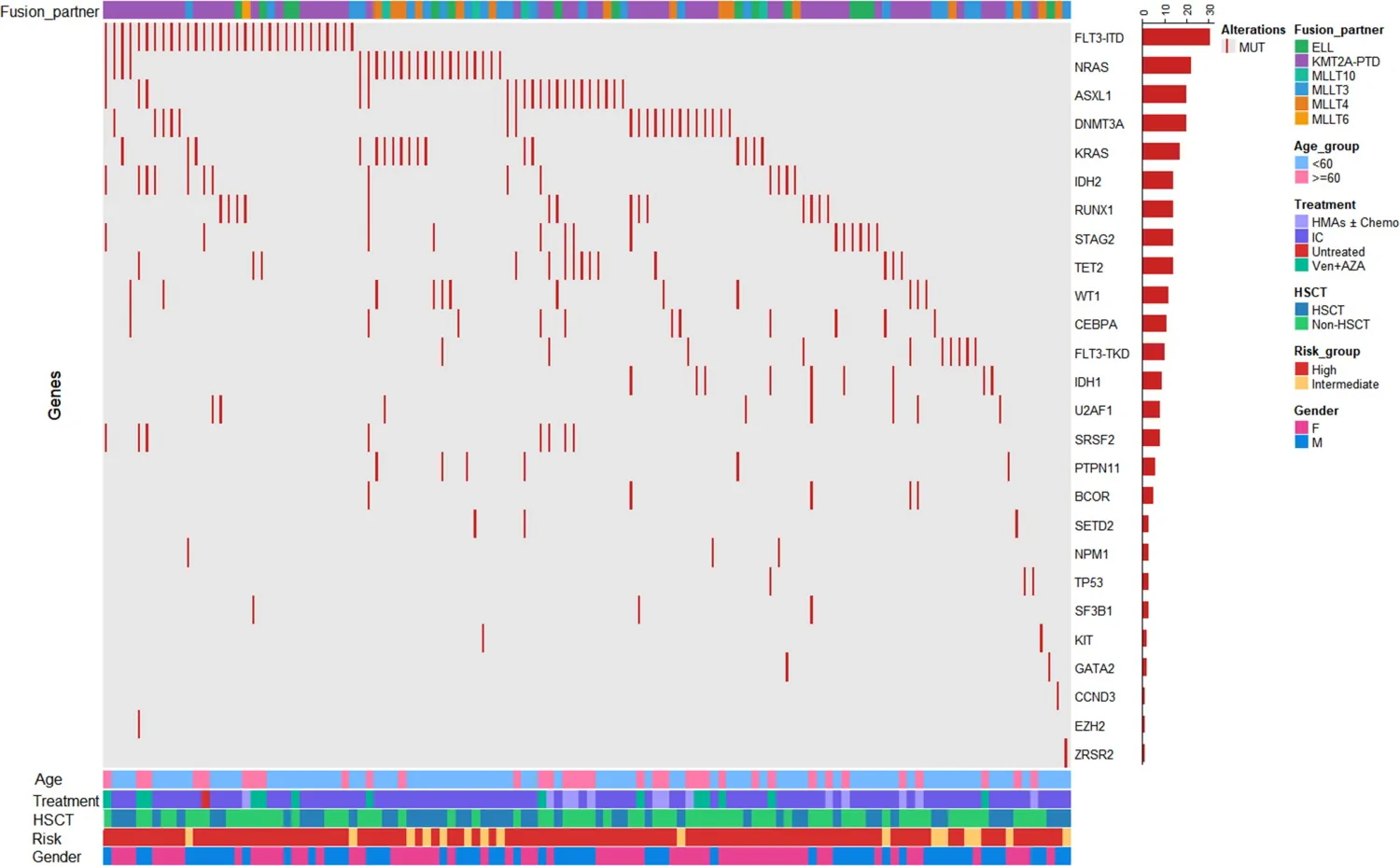
Baseline clinical and genetic features of patients with *KMT2A*-altered AML. Distribution of *KMT2A* aberration subtypes, concurrent mutations, treatment regimen, and ELN-2022 risk stratification in the study cohort.

**Figure 2.**
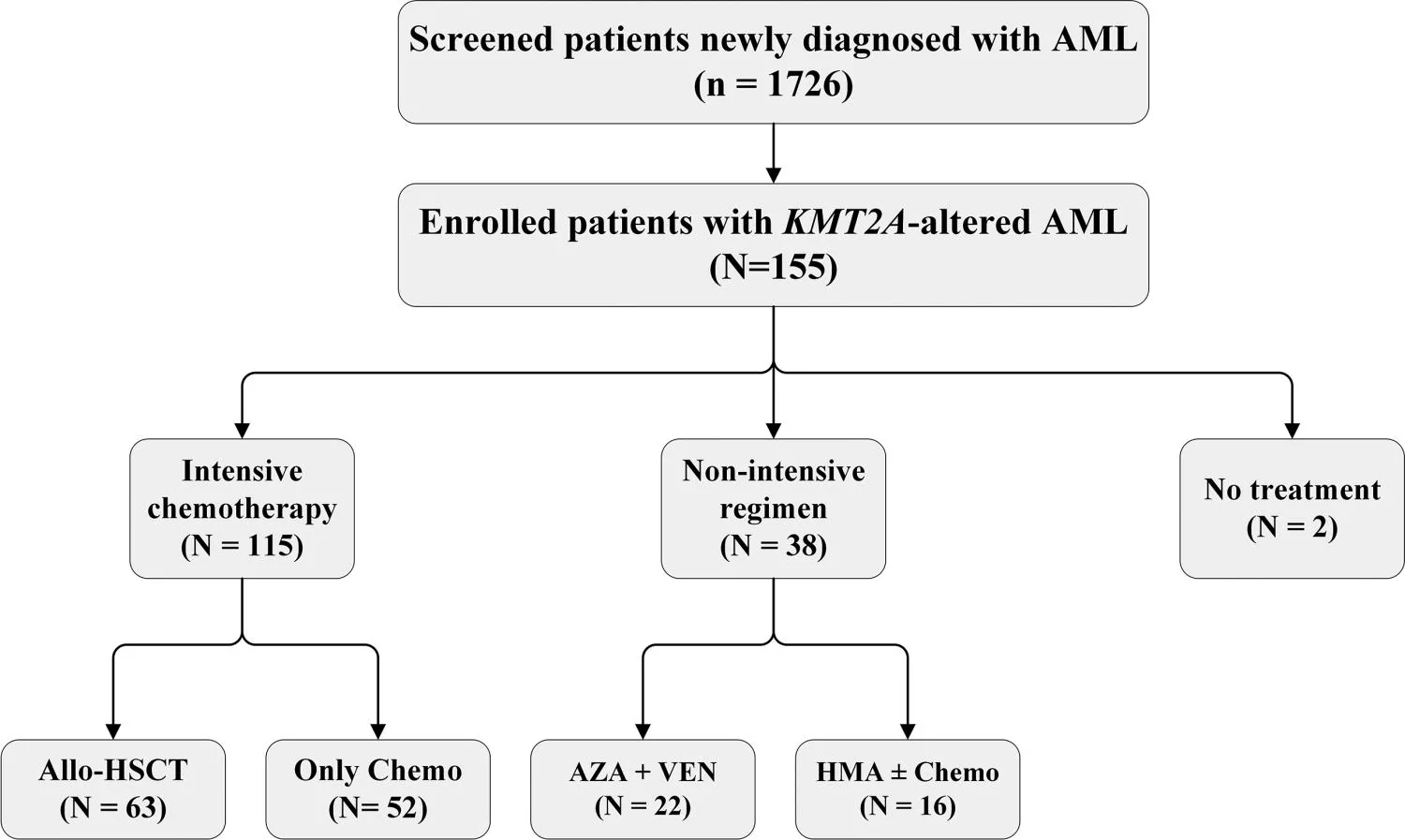
Patient disposition flowchart. Screening, enrolment, and treatment allocation of newly diagnosed adult patients with *KMT2A*-altered acute myeloid leukemia (AML).

**Table 1.** Baseline clinical and genetic characteristics of the enrolled 155 adult patients with KMT2A-altered AML.

Parameters	All Patients (*N* = 155)
Median age, years (range)	48 (14–79)
**Sex, n (%)**	
Male	78 (50.3)
Female	77 (49.7)
**Disease status, n (%)**	
De novo AML	135 (87.1)
Secondary AML (s-AML)	8 (5.2)
Therapy-related AML (t-AML)	12 (7.7)
Median WBC count, ×10^9^/L (range)	13.0 (0.2–239.1)
Median HB, g/L (range)	73 (25–161)
Median PLT count, ×10^9^/L (range)	45 (5–399)
Median serum LDH, IU/L (range)	392 (52–10234)
Median BM blast proportion, % (range)	73.5 (16.5–98.5)
***KMT2A* alteration subtype, n (%)**	
*KMT2A-PTD*	65 (41.9)
*KMT2A::MLLT3*	38 (24.5)
*KMT2A::MLLT4*	23 (14.8)
*KMT2A::ELL*	18 (11.6)
*KMT2A::MLLT10*	9 (5.8)
*KMT2A::MLLT6*	2 (1.3)
**Co-occurring gene mutations, n (%)**	
*FLT3-ITD*	31 (20.0)
*NRAS*	22 (14.2)
*ASXL1*	20 (12.9)
*DNMT3A*	20 (12.9)
*KRAS*	17 (11.0)
*IDH2*	14 (9.0)
*RUNX1*	14 (9.0)
*TET2*	14 (9.0)
*STAG2*	14 (9.0)
Complex chromosome karyotype, n (%)	8 (5.2)
**2022 ELN genetic risk classification, n (%)**	
Intermediate risk	30 (19.4)
Adverse risk	125 (80.6)

*Abbreviations***:** AML: acute myeloid leukemia; s-AML: secondary acute myeloid leukemia; t-AML: therapy-related acute myeloid leukemia; WBC: white blood cell; HB: hemoglobin; PLT: platelet; LDH: lactate dehydrogenase; BM: bone marrow; ELN: European LeukemiaNet.

### 3.2. Treatment regimens and clinical efficacy

Two patients died of severe complications before initiating anti-leukemic treatment; therefore, 153 patients received definitive frontline therapy, including 115 patients treated with intensive chemotherapy (IC), 22 patients treated with venetoclax plus hypomethylating agent (VA), and 16 patients treated with low-intensity hypomethylating agent-based regimens. Baseline characteristics differed significantly across the three groups. Patients in the IC group had a significantly younger median age (39 years) compared with the VA group (65 years) and the low-intensity group (67 years, *p* < 0.0001). The VA group harbored the highest proportion of s/t-AML (36.4%), which was significantly higher than that in the IC group (9.6%) and low-intensity group (6.3%, *p* = 0.0051). The median bone marrow blast percentages were comparable between the IC group (74.5%) and VA group (78.5%), both of which were significantly higher than that of the low-intensity group (56.5%, *p* = 0.0402). The low-intensity group had the highest proportion of *KMT2A-PTD* (75.0%), versus 33.9% in the IC group and 54.5% in the VA group (*p* = 0.0031). The prevalence of *FLT3-ITD* mutation was 20.0%, 27.3%, and 0% across the three groups, respectively (*p* = 0.057). No significant intergroup differences were observed in sex distribution, WBC count, LDH level, or complex karyotype proportion. The baseline characteristics of the three treatment groups are detailed in Table 2.

**Table 2.** Baseline characteristics of patients stratified by frontline treatment regimens.

Parameters	IC (*N* = 115)	AZA + VEN (*N* = 22)	HMAs ± Chemo (*N* = 16)	*p*-Value
Median age, years (range)	39 (14–71)	65 (43–79)	67 (60–77)	<0.0001
Male, n (%)	55 (47.8)	11 (50.0)	10 (62.5)	0.5440
s-AML/t-AML, n (%)	11 (9.6)	8 (36.4)	1 (6.3)	0.0051
Median WBC, ×10^9^/L (range)	13.3 (0.4–239.1)	24.3 (1.2–175.7)	4.4 (0.2–203.0)	0.2771
Median LDH, IU/L (range)	382 (52–10234)	466 (147–1984)	302 (111–3313)	0.7200
Median BM blast, % (range)	74.5 (16.5–98.5)	78.5 (18.5–97.5)	56.5 (20.0–93.0)	0.0402
Complex chromosome karyotype, n (%)	5 (4.3)	3 (13.6)	0 (0.0)	0.1579
***KMT2A* alteration subtype, n (%)**				
*KMT2A-PTD*	39 (33.9)	12 (54.5)	12 (75.0)	0.0031
*KMT2A::MLLT3*	34 (29.6)	2 (9.1)	2 (12.5)	0.0759
*KMT2A::MLLT4*	18 (15.7)	4 (18.2)	1 (6.3)	0.5922
*KMT2A::ELL*	15 (13.0)	3 (13.6)	0 (0.0)	0.3537
*KMT2A::MLLT10*	9 (7.8)	0 (0.0)	0 (0.0)	0.3390
*KMT2A::MLLT6*	0 (0)	1 (4.5)	1 (6.3)	0.0605
**Co-occurring mutations, n (%)**				
*FLT3-ITD*	24 (20.9)	6 (27.3)	0 (0.0)	0.0570
*NRAS*	20 (17.4)	2 (9.1)	0 (0.0)	0.1584
*ASXL1*	11 (9.6)	5 (22.7)	4 (25.0)	0.0522
*DNMT3A*	12 (10.4)	5 (22.7)	3 (18.8)	0.1736
**2022 ELN risk stratification, n (%)**				
Intermediate risk	26 (22.6)	2 (9.1)	2 (12.5)	0.3128
Adverse risk	89 (77.4)	20 (90.9)	14 (87.5)	–

*Abbreviations*. IC: intensive chemotherapy; AZA: azacitidine; VEN: venetoclax; HMAs: hypomethylating agents; Chemo: chemotherapy; AML: acute myeloid leukemia; WBC: white blood cell; LDH: lactate dehydrogenase; BM: bone marrow; ELN: European LeukemiaNet. Two patients without any anti-leukemic treatment were not enrolled in this table.

The median follow-up duration for the entire treated cohort was 38.8 months. The overall complete remission (CR) rate was 70.7%, the partial remission (PR) rate was 8.2%, and the overall response rate (ORR) reached 78.9%. Treatment-related mortality during induction therapy occurred in 6 patients (3.9%). For patients who achieved initial CR, the median relapse-free survival (RFS) of the entire cohort was 12.4 months (Figure 3A), and the median overall survival (OS) was 16.4 months (Figure 3B). Stratified by frontline regimens, the IC and VA groups showed comparable CR rates (73.5% vs. 75.0%), both numerically higher than that of the low-intensity group (42.9%, *p* = 0.0634). Notably, the IC group achieved a significantly higher molecular MRD negativity rate (47.8%) than the VA group (20.0%) and low-intensity group (14.3%, *p* = 0.0045). Survival analysis revealed no significant difference in median RFS across the three groups (IC: 13.5 months, VA: 5.3 months, low-intensity: 19.6 months, *p* = 0.0980, Figure 3C). However, the IC group exhibited significantly longer median OS (22.4 months) compared with the VA group (10.2 months) and low-intensity group (7.4 months, *p* = 0.0034, Figure 3D). Detailed treatment outcomes are summarized in Table 3.

**Figure 3.**
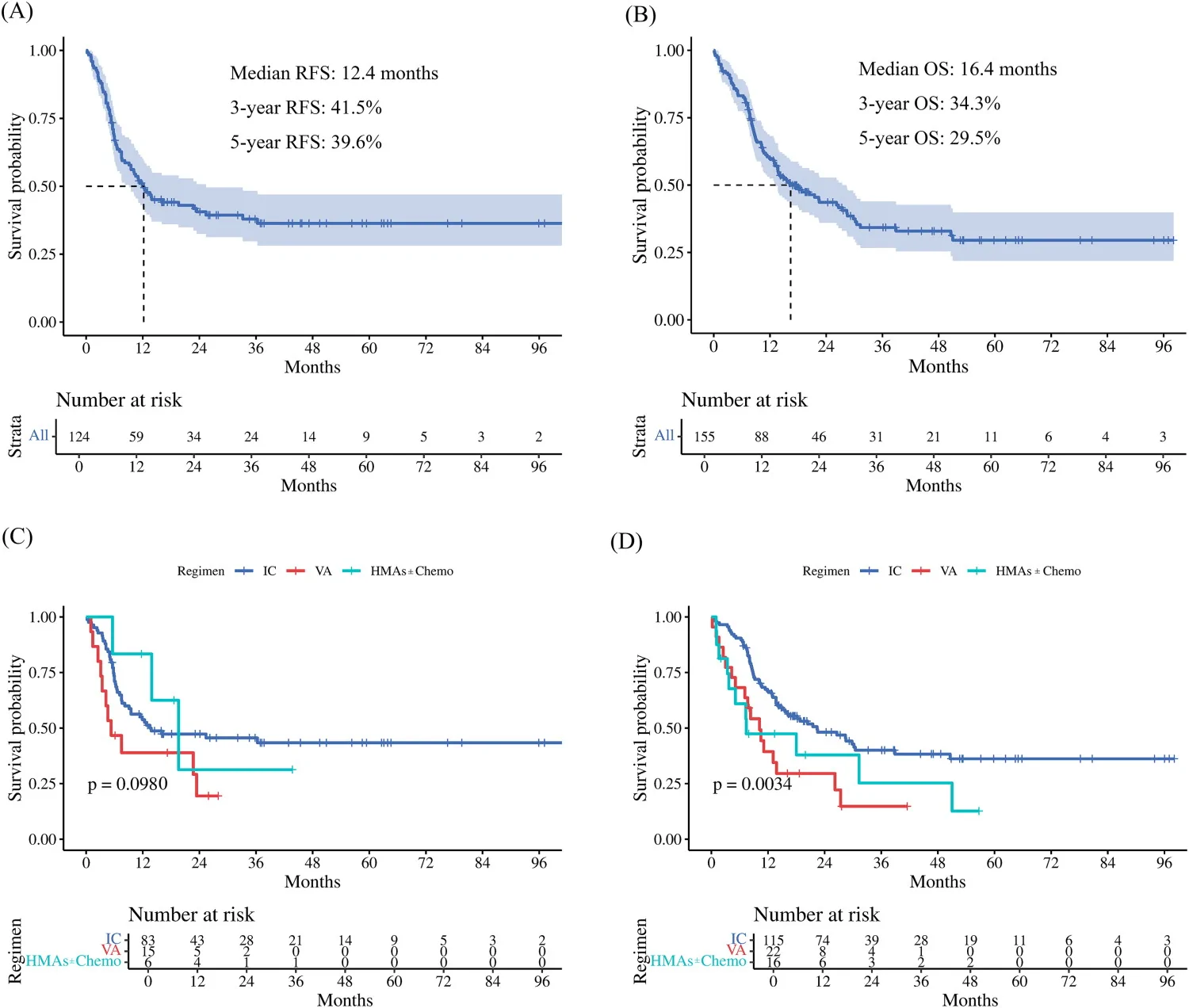
Survival stratified by frontline treatment regimens. Kaplan-Meier curves for (A) relapse-free survival (RFS) and (B) overall survival (OS) of the whole treated cohort. Comparison of (C) RFS and (D) OS across intensive chemotherapy (IC), venetoclax-based (VA), and low-intensity regimen groups.

**Table 3.** Treatment responses and survival outcomes of patients receiving different induction regimens.

Parameters	Total Treated Patients (*N* = 153)	IC (*N* = 115)	AZA + VEN (*N* = 22)	HMAs ± Chemo (*N* = 16)	*p*-Value
**Treatment response, n (%)**					
CR/CRi	104 (70.7)	83 (73.5)	15 (75.0)	6 (42.9)	0.0634
PR	12 (8.2)	10 (8.9)	0 (0.0)	2 (14.3)	0.2575
ORR	116 (78.9)	93 (82.3)	15 (75.0)	8 (57.1)	0.0880
Allo-HSCT performed, n (%)	63 (41.2)	63 (54.8)	0 (0.0)	0 (0.0)	<0.0001
Flow cytometric phenotypic MRD negativity, n (%)	108 (73.5)	91 (80.5)	13 (65.0)	4 (28.6)	0.0002
Molecular MRD negativity *, n (%)	60 (40.8)	54 (47.8)	4 (20.0)	2 (14.3)	0.0045
Induction mortality, n (%)	6 (3.9)	2 (1.7)	2 (9.1)	2 (12.5)	0.0423
**Survival of all treated patients**					
Median RFS, months	12.4	13.5	5.3	19.6	0.0980
Median OS, months	16.4	22.4	10.2	7.4	0.0034
**Survival of patients without Allo-HSCT**					
Median RFS, months	6.3	6.1	5.3	19.6	0.0620
Median OS, months	9.2	9.2	10.2	7.4	0.5969

*Abbreviations*. IC: intensive chemotherapy; AZA: azacitidine; VEN: venetoclax; HMAs: hypomethylating agents; Chemo: chemotherapy; CR: complete remission; CRi: complete remission with incomplete hematologic recovery; PR: partial remission; ORR: overall response rate; MRD: minimal residual disease; Allo-HSCT: allogeneic hematopoietic stem cell transplantation; RFS: relapse-free survival; OS: overall survival. * A small subset of patients lacked valid molecular MRD data due to early death during induction or inconsistent post-treatment genetic monitoring. Percentages for response and MRD rates were calculated among evaluable patients (*n* = 147), whereas those for Allo-HSCT and induction mortality were based on all treated patients (*n* = 153).

Among all treated patients, 63 (41.2%) received Allo-HSCT during the disease course, 108 (73.5%) achieved negative MRD by flow-cytometric immunophenotyping, and 60 (40.8%) achieved negative *KMT2A*-based molecular MRD. The baseline clinical features and disease status of these patients are summarized in Supplementary Table 2. Patients with flow-cytometric MRD negativity had a median OS of 30.5 months, which was significantly superior to 8.7 months in flow-MRD-positive patients and 3.8 months in non-remission patients (*p* < 0.0001, Figure 4A). Further subgroup analysis among flow-MRD-negative patients demonstrated that those who achieved concurrent *KMT2A* molecular MRD negativity had not reached median RFS and OS during follow-up (Figure 4B, 4C), which were markedly better than the median RFS of 8.2 months and median OS of 15.6 months in patients with persistent *KMT2A* molecular MRD positivity (both *p* < 0.0001).

**Figure 4.**
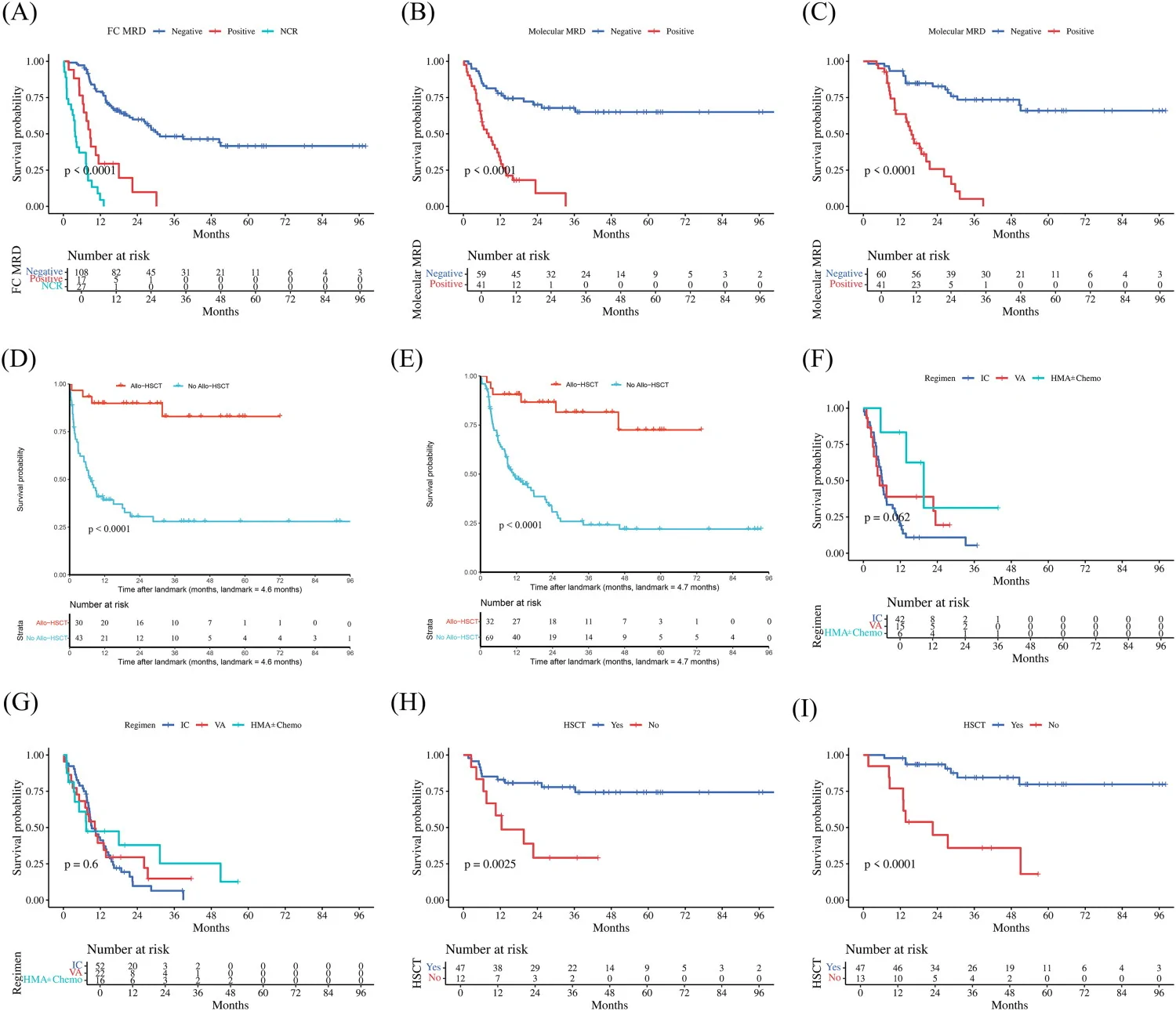
Prognostic analyses of MRD status and allogeneic hematopoietic stem cell transplantation (Allo-HSCT). (A) OS stratified by flow-cytometric MRD and remission status. (B-C) RFS and OS among flow-MRD-negative patients stratified by *KMT2A* molecular MRD. (D-E) Landmark analysis of RFS and OS comparing patients with versus without Allo-HSCT. The landmark time was set at the median time from diagnosis to transplantation (4.6 months). Only patients alive and free of events at this landmark time were included, and survival was calculated starting from the landmark time point. (F-G) RFS and OS for three induction groups after excluding transplant recipients. (H-I) RFS and OS according to Allo-HSCT among patients achieving *KMT2A* molecular MRD negativity.

### 3.3. Prognostic impact of allogeneic hematopoietic stem cell transplantation

Of the entire cohort, 63 patients received Allo-HSCT during their disease course, including 55 transplanted in first CR (CR1), 2 in partial remission (PR), 3 with primary refractory disease, and 3 with relapsed disease. One patient underwent a second transplantation after post-transplant relapse. During follow-up, 19 transplant recipients died, with 6 deaths attributed to leukemic relapse and 13 to transplant-related complications.

Patients receiving Allo-HSCT had not reached median RFS and OS, whereas non-transplant patients exhibited a median RFS of 6.3 months and a median OS of 9.2 months (Figure 4D, 4E). Given that all transplant recipients were from the IC group, we further compared survival outcomes among the three regimens after excluding transplanted patients. The results showed no significant intergroup differences in RFS (6.1 months vs. 5.3 months vs. 19.6 months, *p* = 0.0620) and OS (9.2 months vs. 10.2 months vs. 7.4 months, *p* = 0.5969, Figure 4F, 4G). Importantly, 74.6% of transplant recipients achieved *KMT2A* molecular MRD negativity, which was substantially higher than 14.4% in non-transplant patients (*p* < 0.0001). Furthermore, among patients with molecular MRD negativity, those who received Allo-HSCT achieved significantly superior RFS and OS compared with non-transplant counterparts (Figure 4H, 4I).

### 3.4. Dynamics of *KMT2A* molecular MRD before and after Allo-HSCT

Among the 63 patients who underwent Allo-HSCT, we further evaluated changes in *KMT2A* molecular MRD status from pre-transplant to post-transplant follow-up. As visualized in Supplementary Figure 1, all 18 patients with pre-transplant *KMT2A* molecular MRD negativity retained molecular negativity after transplantation. Of the 45 patients with pre-transplant *KMT2A* molecular MRD positivity, 29 (64.4%) converted to *KMT2A* MRD negativity post-transplant, whereas 16 (35.6%) remained MRD-positive.

### 3.5. Univariate and multivariate prognostic analyses

Univariate survival analysis was performed to evaluate the prognostic impacts of clinical and molecular variables, including age, sex, peripheral blood indices, LDH level, *KMT2A* aberration subtype, AML etiology, bone marrow blast proportion, and ELN risk stratification (Figure 5). To further evaluate potential non-linear relationships between continuous clinical variables and survival outcomes, restricted cubic spline (RCS) Cox regression was conducted as a sensitivity analysis in 140 patients with complete data for age, LDH, peripheral WBC count and bone marrow blast percentage. Age was significantly associated with survival (*p* = 0.0003), while no statistically significant non-linear relationship was observed (P for non-linearity = 0.0639). LDH showed no significant association with survival (*p* = 0.9808), with no evidence of non-linearity (P for non-linearity = 0.8457). The association between WBC count and survival was not statistically significant (*p* = 0.0625), and no significant non-linear relationship was detected (P for non-linearity = 0.0769). Bone marrow blast percentage was not associated with survival (*p* = 0.4905), and no non-linear association was identified (P for non-linearity = 0.2967). Collectively, these RCS analyses did not demonstrate statistically significant non-linear relationships for any of the four continuous variables, supporting our primary analysis using the predefined age cutoff of 60 years. The hazard ratio curves are presented in Supplementary Figure 2.

**Figure 5.**
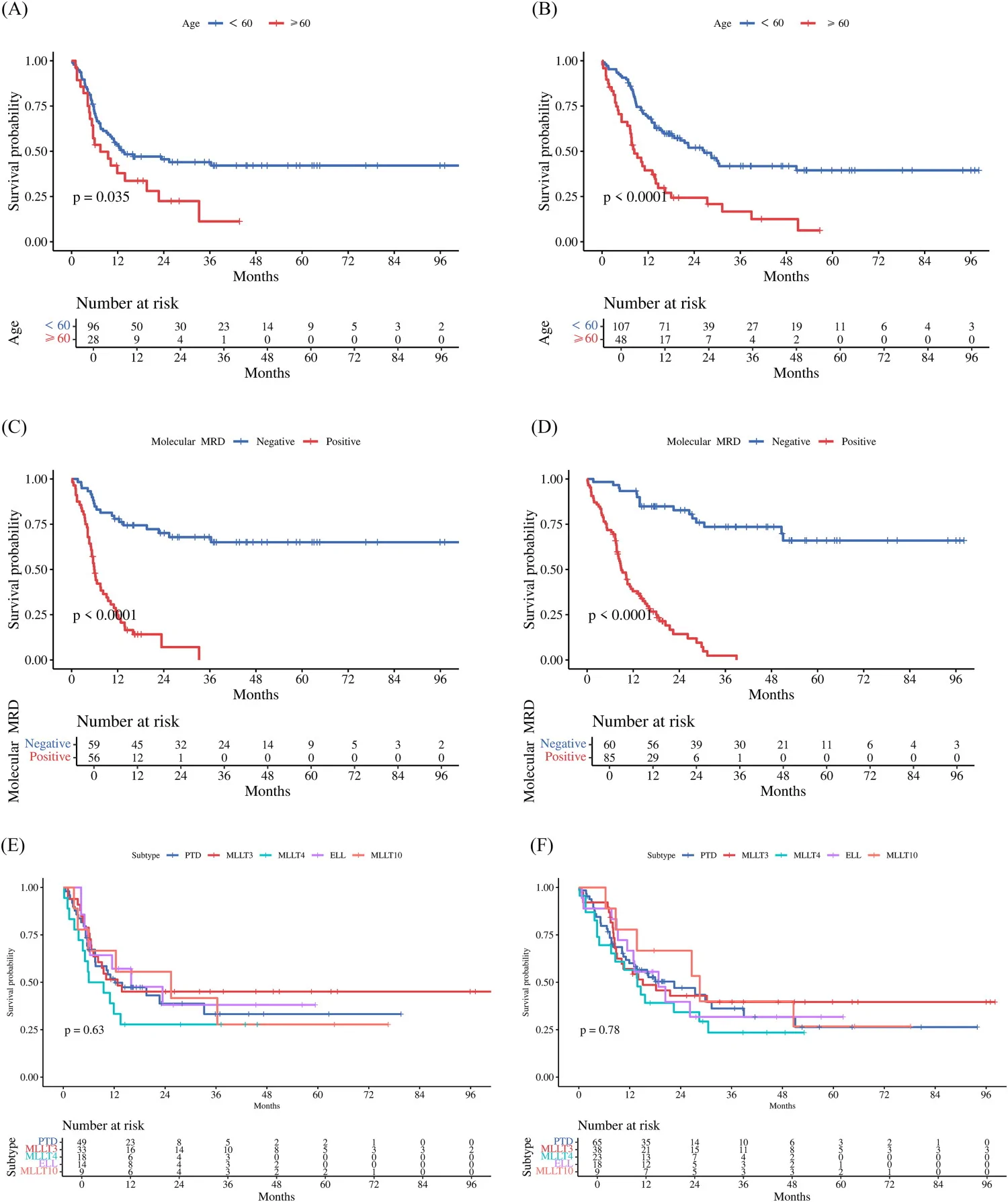
Univariate Kaplan–Meier prognostic analysis of key clinical and genetic factors in *KMT2A*-altered AML. Kaplan–Meier curves for (A, B) RFS and OS stratified by age; (C, D) RFS and OS stratified by *KMT2A* molecular MRD status; (E, F) RFS and OS across different *KMT2A* aberration subtypes. Abbreviations: Allo-HSCT, allogeneic hematopoietic stem cell transplantation; IC, intensive chemotherapy; MRD, minimal residual disease; OS, overall survival; RFS, relapse-free survival; VA, venetoclax plus hypomethylating agent; HMA, hypomethylating agent; Chemo, chemotherapy.

The results demonstrated that advanced age, MRD status, and Allo-HSCT were significant prognostic factors for both RFS and OS, whereas other clinical and molecular variables showed no consistent prognostic significance. Patients aged ≥60 years had inferior survival, with a median RFS of 5.6 months and a median OS of 8.4 months, which were significantly shorter than those in younger patients (RFS: 13.5 months, *p* = 0.035; OS: 26.6 months, *p* < 0.0001). Patients with molecular MRD negativity had unreached median RFS and OS, which were significantly better than those with persistent MRD positivity (RFS: 5.9 months, OS: 9.2 months, both *p* < 0.0001). Similarly, transplant recipients achieved significantly improved RFS and OS compared with non-transplant patients (both *p* < 0.0001).

Multivariate Cox proportional hazards regression analysis was further performed to identify independent prognostic factors (Table 4). Persistent molecular MRD positivity emerged as a powerful independent adverse prognostic factor for inferior RFS (HR = 4.780, 95% CI: 2.205–10.360, *p* < 0.001) and OS (HR = 4.802, 95% CI: 2.415–9.545, *p* < 0.001). The absence of Allo-HSCT also independently predicted poor RFS (HR = 3.734, 95% CI: 1.671–8.345, *p* = 0.001) and OS (HR = 3.376, 95% CI: 1.660–6.862, *p* = 0.001). After multivariate adjustment, advanced age lost independent prognostic value, indicating that the prognostic discrepancy related to age was largely confounded by treatment accessibility and transplantation status.

**Table 4.** Multivariate Cox regression analysis for prognostic factors of RFS and OS.

Variables	Relapse-Free Survival (RFS)		Overall Survival (OS)	
	HR (95% CI)	*p*-Value	HR (95% CI)	*p*-Value
Age ≥ 60 years	0.714 (0.384–1.328)	0.287	0.995 (0.620–1.597)	0.983
Without Allo-HSCT	3.734 (1.671–8.345)	0.001	3.376 (1.660–6.862)	0.001
Persistent molecular MRD positivity	4.780 (2.205–10.360)	<0.001	4.802 (2.415–9.545)	<0.001

Abbreviations: HR: hazard ratio; CI: confidence interval; Allo-HSCT: allogeneic hematopoietic stem cell transplantation; MRD: minimal residual disease.

To account for guarantee-time bias related to Allo-HSCT, we further performed sensitivity analysis using time-dependent multivariate Cox regression with a start-stop data structure, where Allo-HSCT was modelled as a time-updated covariate. For RFS, Allo-HSCT showed a trend toward reduced relapse risk but was not statistically significant (HR = 0.536, 95%CI: 0.233–1.237, *p* = 0.144). Molecular MRD positivity was a strong independent adverse prognostic factor (HR = 9.850, 95%CI: 4.441– 21.850, *p* < 0.001). Age was not independently associated with RFS (HR = 0.993, 95%CI: 0.977–1.010, *p* = 0.414). For OS, molecular MRD positivity remained a dominant independent adverse biomarker (HR = 8.343, 95%CI: 4.196–16.589, *p* < 0.001). Allo-HSCT exhibited a trend toward lower mortality risk without reaching statistical significance (HR = 0.586, 95%CI: 0.304–1.129, *p* = 0.110). Age had no significant impact on OS (HR = 1.004, 95%CI: 0.992–1.017, *p* = 0.520). Results of the time-dependent Cox models are presented in Table 5.

**Table 5.** Time-dependent multivariate Cox regression analyses for relapse-free survival (RFS) and overall survival (OS) in patients with KMT2A-altered AML.

Variable	HR	95% CI	*p* Value
**RFS**			
Allo-HSCT (time-dependent)	0.536	0.233–1.237	0.144
Age, per 1-year increase	0.993	0.977–1.010	0.414
Molecular MRD positive (vs negative)	9.850	4.441–21.850	<0.001
**OS**			
Allo-HSCT (time-dependent)	0.586	0.304–1.129	0.110
Age, per 1-year increase	1.004	0.992–1.017	0.520
Molecular MRD positive (vs negative)	8.343	4.196–16.589	<0.001

Abbreviations: HR: hazard ratio; CI: confidence interval; RFS: relapse-free survival; OS: overall survival; Allo-HSCT: allogeneic hematopoietic stem cell transplantation; MRD: minimal residual disease.

## 4. Discussion

*KMT2A*-altered AML is universally recognized as a high-risk AML subtype characterized by high refractoriness and relapse risk, with unsatisfactory long-term survival, and no unified optimal treatment strategy has been established for this population to date [3,6,16]. In this large real-world single-center cohort of 155 adult patients with newly diagnosed *KMT2A*-altered AML, we systematically clarified the hierarchical prognostic value of frontline induction regimens, dual MRD status (flow-cytometric phenotypic and *KMT2A* molecular MRD), and Allo-HSCT consolidation. Distinct from previous studies that merely compared the efficacy of different induction regimens or simply identified prognostic risk factors, our study provides a hypothesis-generating interpretation: the survival advantage observed in the IC group may be partly explained by sustained *KMT2A* molecular MRD clearance associated with Allo-HSCT, rather than the inherent superiority of intensive induction therapy. We acknowledge important baseline imbalances across treatment groups, including marked age differences and the fact that no patients in non-intensive treatment arms received Allo-HSCT, which limits causal inference from this retrospective observational analysis. These findings may offer novel real-world evidence for MRD-guided transplantation decision-making in high-risk *KMT2A*-altered AML.

Consistent with previous clinical reports [6,7], our cohort confirmed the poor prognosis of *KMT2A*-altered AML, with an overall ORR of 78.9% and a median OS of only 16.4 months. Prior studies have demonstrated that intensive chemotherapy, VA combination regimens, and low-intensity regimens yield heterogeneous efficacy in high-risk AML, with IC regimens generally achieving higher morphological remission rates in young and fit patients, while VA regimens are more applicable for elderly and unfit populations. In line with these findings, our study showed comparable CR rates between IC and VA groups (73.5% vs. 75.0%), both numerically higher than that of low-intensity therapy. However, previous literature often attributed the survival differences among treatment groups to the advantages of intensive induction regimens. By contrast, our stratified analysis eliminated the confounding effect of Allo-HSCT and yielded a novel conclusion: after excluding transplant recipients, RFS and OS were statistically comparable among the three non-transplant subgroups, indicating that frontline induction regimens themselves cannot bring inherent long-term survival benefits for *KMT2A*-altered AML.

MRD status has been validated as the most critical dynamic prognostic biomarker for AML relapse and survival in multiple studies [9,10], but most existing research only focused on conventional flow-cytometric phenotypic MRD, and few studies have systematically compared the prognostic value of flow MRD and *KMT2A* gene-specific molecular MRD in *KMT2A*-altered AML. Our study innovatively performed hierarchical stratification based on dual MRD indicators and found that patients with flow-cytometric MRD negativity achieved a markedly superior median OS of 30.5 months, far exceeding that of flow MRD-positive and non-remission patients. More importantly, even among patients with phenotypic MRD negativity, persistent *KMT2A* molecular residual lesions still led to poor long-term outcomes; patients with concurrent molecular MRD negativity had unreached RFS and OS during follow-up, which were significantly better than those with residual *KMT2A* alteration. These findings further supplement and refine previous research conclusions [6], confirming that *KMT2A* molecular MRD represents a more rigorous and reliable prognostic indicator than traditional flow phenotypic MRD, and deep molecular remission should be regarded as the core therapeutic endpoint for *KMT2A*-altered AML.

Allo-HSCT represents the curative backbone for high-risk AML [17,18]. Although survival benefits of transplantation have been documented in patients with *KMT2A*-altered AML [6,19,20], the molecular mechanism underlying such prognostic improvement, particularly the quantitative link between Allo-HSCT-driven *KMT2A* molecular MRD clearance and long-term clinical outcomes, remains insufficiently validated in real-world cohorts. Several studies have suggested that the survival benefit from Allo-HSCT is largely mediated by graft-versus-leukemia activity that eradicates residual leukemic clones [21,22]. In our cohort, 74.6% of transplant recipients achieved sustained *KMT2A* molecular MRD negativity, which was significantly higher than the 14.4% rate in non-transplant patients. Moreover, even in patients who achieved molecular MRD negativity, those receiving Allo-HSCT still obtained superior RFS and OS, fully verifying that Allo-HSCT exerts a powerful residual clone clearance effect, which cannot be replaced by conventional induction and consolidation therapy. Given that all transplant recipients in our cohort received IC-based induction, our findings provide a plausible mechanistic interpretation for the observed survival advantage among IC-treated patients: intensive induction therapy enables morphological remission and renders patients eligible for subsequent Allo-HSCT, whereas Allo-HSCT is the primary intervention that eradicates *KMT2A*-mutated clones and transforms transient morphological remission into sustained molecular remission, leading to improved long-term outcomes.

Recent advances in transplant platforms have expanded the safety and accessibility of allo-HSCT. Post-transplant cyclophosphamide (PTCy)-based GVHD prophylaxis has emerged as a flexible strategy across matched and mismatched donor settings, effectively lowering GVHD-related morbidity while preserving graft-versus-leukemia activity, which has reshaped modern transplant practice for AML patients [23–26]. Meanwhile, validated predictive models for post-transplant AML relapse have been developed to integrate pre-transplant disease status, molecular features and transplant-related variables, facilitating individualized risk stratification and post-transplant surveillance [27,28]. In our cohort, *KMT2A*-specific molecular MRD represents a strong prognostic biomarker that could be incorporated together with these modern transplant risk models to refine relapse prediction for patients with *KMT2A*-altered AML undergoing Allo-HSCT.

Consistent with multiple previous AML prognostic studies [29,30], univariate analysis in our cohort showed that advanced age was a significant adverse prognostic factor for RFS and OS. However, after multivariate adjustment, age lost independent prognostic significance. This phenomenon indicates that the poor prognosis of elderly patients with *KMT2A*-altered AML is not caused by age itself, but by clinical confounding factors such as poor physical tolerance, limited access to intensive induction therapy, and low transplantation feasibility. In addition, we observed that neither distinct *KMT2A* aberration subtypes nor co-occurring gene mutations represented independent prognostic factors in our cohort. These findings suggest that, in the context of post-treatment monitoring and transplant intervention, the prognostic influence of baseline genetic heterogeneity may be outweighed by molecular MRD status after therapy. This observation adds important evidence for subgroup-specific prognostic analyses for patients with *KMT2A*-altered AML.

This study has several inherent limitations that should be acknowledged. First, this is a single-center retrospective study with inevitable selection bias, and the non-randomized treatment allocation may affect the efficacy comparison of different induction regimens. Second, although the overall sample size is relatively large for rare *KMT2A*-altered AML, the sample size of some stratified subgroups is limited. Third, the dynamic MRD monitoring time points were not fully unified, and continuous longitudinal MRD changes were not analyzed. In addition, age was dichotomized at 60 years in the primary analysis; we further explored potential non-linear associations of continuous clinical variables with survival using restricted cubic spline Cox models. No statistically significant non-linear relationships were identified. Therefore, future prospective multicenter studies with standardized MRD monitoring protocols are required to further validate our findings.

In *KMT2A*-altered AML, sustained *KMT2A* molecular MRD negativity is the key determinant of favorable long-term survival. Allo-HSCT is associated with higher rates of *KMT2A* molecular MRD clearance and represents a potential consolidation strategy to achieve deep molecular remission. Compared with conventional flow cytometric phenotypic MRD, gene-specific *KMT2A* molecular MRD provides superior prognostic discrimination for deep remission and clinical outcomes. Strategies targeting sustained molecular MRD negativity *via* Allo-HSCT may help eradicate residual leukemic clones, though this association should be interpreted cautiously due to guarantee-time bias and inherent limitations of this retrospective cohort.

## Supplementary Material

Supplemental Material

Supplementary Table 2.docx

Supplementary Figure 1.jpg

Supplementary File 1.docx

Supplementary Figure 2.jpg

Supplementary Table 1.docx

## Data Availability

The data supporting the findings of this study are available from the corresponding author upon reasonable request. Further enquiries can be directed to the corresponding author *via* email.
