# Supplementary material for: Allogeneic hematopoietic stem cell transplantation is associated with improved long-term survival and molecular MRD clearance in KMT2A-altered AML
Source: Ann Med. 2026 Nov 12;58(1):2758994. doi: 10.1080/07853890.2026.2758994 (PMC13761046; doi:10.1080/07853890.2026.2758994)
Supplement: Supplementary file 1 [file IANN_A_2758994_SM1010.docx]

Supplementary Figures

Supplementary Figure 1. Alluvial diagram illustrating transitions of KMT2A molecular MRD status before and after allogeneic hematopoietic stem cell transplantation (Allo-HSCT) among the 63 transplanted patients. Of patients with pre-transplant KMT2A MRD negativity (n=18), all sustained molecular remission after transplantation. Among patients with pre-transplant KMT2A MRD positivity (n=45), 29 converted to MRD negativity and 16 remained MRD positive post-transplant. MRD, minimal residual disease.

Supplementary Figure 2. Restricted cubic spline analyses for the continuous relationship between baseline clinical parameters and hazard ratio of overall survival among KMT2A-altered AML patients. (A) Age (red dashed line = 60 years). (B) White blood cell count. (C) Lactate dehydrogenase (LDH). (D) Bone marrow blast percentage. Blue solid lines denote hazard ratios, and grey shaded regions show 95% confidence intervals.
