# Supplementary material for: Allogeneic hematopoietic stem cell transplantation is associated with improved long-term survival and molecular MRD clearance in KMT2A-altered AML
Source: Ann Med. 2026 Nov 12;58(1):2758994. doi: 10.1080/07853890.2026.2758994 (PMC13761046; doi:10.1080/07853890.2026.2758994)
Supplement: Supplementary file 2 [file IANN_A_2758994_SM1175.docx]

| Supplementary Table 1. The details of initial treatment protocols administered in this study. | | |
| --- | --- | --- |
| Regimen | Number | Drugs |
| IC |  |  |
| IA protocol | 98 | 4-week cycle: idarubicin at a dose of 8 to 10 mg/m² was administered intravenously once daily for a total of 3 consecutive days; cytarabine at a dose of 100 mg/m² was given by intravenous infusion every 12 hours, continuing for 7 consecutive days. |
| DA protocol | 17 | 4-week cycle: daunorubicin at a dose of 45 to 60 mg/m² was administered intravenously once daily for a total of 3 consecutive days; cytarabine at a dose of 100 mg/m² was given by intravenous infusion every 12 hours, continuing for 7 consecutive days. |
| AZA + VEN | 22 | 4-week cycle: azacitidine: 75 mg/m^2^, subcutaneous injection once a day from days 1 to 7; venetoclax: 100 mg on day 1, 200 mg on day 2, 400 mg from days 3 to 28, oral administration once a day. |
|  |  | For patients intolerant to venetoclax or experiencing significant hematological toxicity, the dosing may be adjusted to 200 or 300 mg per day, and venetoclax was temporarily held in patients who developed neutropenia complicated by severe infection. |
| HMAs ± chemotherapy | 16 |  |
| AZA monotherapy | 4 | Azacitidine at a dose of 75 mg/m² was administered subcutaneously once daily for a consecutive period of 7 days. |
| HMAs + low-dose chemotherapy | 12 | 4-week cycle: decitabine, 15-20 mg/m^2^, intravenous injection once a day from days 1 to 5 or azacitidine, 75 mg/m^2^, subcutaneous injection once a day from days 1 to 7; aclacinomycin, 10 mg per day, intravenous injection once a day from days 3 to 6 or homoharringtonine 2 mg per day, intravenous injection once a day from days 3 to 9; cytarabine: 10 mg/m^2^, intravenous injection twice a day from days 3 to 9; G-CSF: 5 μg/kg, subcutaneous injection once a day from day 0 until neutrophil recovery. G-CSF was withheld when WBC count ≥20×10^9^/L. |
| IC: intensive chemotherapy; HMAs: hypomethylating agents; AZA: azacitidine; DEC: decitabine; VEN: venetoclax. Chemotherapy cycles were adjusted according to patients’ bone‑marrow hematopoietic recovery and concurrent infectious complications. | | |
