# Supplementary material for: Allogeneic hematopoietic stem cell transplantation is associated with improved long-term survival and molecular MRD clearance in KMT2A-altered AML
Source: Ann Med. 2026 Nov 12;58(1):2758994. doi: 10.1080/07853890.2026.2758994 (PMC13761046; doi:10.1080/07853890.2026.2758994)
Supplement: Supplementary file 3 [file IANN_A_2758994_SM1177.docx]

**Quantification of *KMT2A* fusion transcripts and *KMT2A-PTD***

Quantitative detection of multiple *KMT2A* fusion transcripts and *KMT2A-PTD* was conducted by KangSheng Global (Beijing, China). **Reverse transcription quantitative real-time PCR (RT-qPCR)** was adopted for transcript quantification.

Briefly, total RNA was extracted from EDTA-anticoagulated bone marrow specimens using TaKaRa RNAiso Plus (Cat# 9109, Takara Bio). RNA was reverse-transcribed into complementary DNA (cDNA) with PrimeScript™ RT reagent Kit (Cat# RR037A, Takara Bio). PCR amplification mixtures were prepared using Taqman Fast Advanced Master Mix (Cat# 4444965, Life Technologies). Target-specific primers and HPLC-purified probes (General Biol, Anhui, China) were added for amplification of *KMT2A* fusion transcripts and *KMT2A-PTD*. Amplification and real-time fluorescence signal acquisition were performed on the ABI 7500 Real-Time PCR System (Life Technologies).

The detailed reagents and equipment used in the assay were listed below:

| **Reagent / Equipment** | **Catalog No.** | **Manufacturer** | **Function** |
| --- | --- | --- | --- |
| TaKaRa RNAiso Plus | 9109 | TAKARA | Total RNA extraction |
| PrimeScript^TM^ RT reagent Kit | RR037A | TAKARA | Reverse transcription of RNA to cDNA |
| Taqman Fast Advanced Master Mix | 4444965 | Life Technologies Co.,Ltd. | Master mix for RT-qPCR amplification |
| HPLC-purified primers | N/A | General Biol (Anhui) Co., Ltd. | Target-specific PCR primers |
| HPLC-purified probes | N/A | General Biol (Anhui) Co., Ltd. | Taqman fluorescent probes |
| ABI 7500 Real-Time PCR System | ABI7500 | Life Technologies Co.,Ltd. | RT-qPCR amplification and data acquisition |

Digital PCR was available as an alternative quantitative platform in the laboratory, but RT-qPCR was used for all patient samples in this cohort.
