# Supplementary material for: Allogeneic hematopoietic stem cell transplantation is associated with improved long-term survival and molecular MRD clearance in KMT2A-altered AML
Source: Ann Med. 2026 Nov 12;58(1):2758994. doi: 10.1080/07853890.2026.2758994 (PMC13761046; doi:10.1080/07853890.2026.2758994)
Supplement: Supplementary file 4 [file IANN_A_2758994_SM1180.docx]

| Supplementary Table 2. Characteristics of the 63 patients who underwent Allo-HSCT. | |
| --- | --- |
| **Variable** | **Results** |
| Total, n | 63 |
| Median age, year (range) | 37 (14-59) |
| Disease status, n (%) |  |
| CR | 55 (87.3) |
| PR | 2 (3.2) |
| Relapse | 3 (4.8) |
| Refractory | 3 (4.8) |
| Pre-transplant MRD status, n (%) |  |
| Flow-MRD negative | 35 (55.6) |
| *KMT2A* molecular MRD negative | 18 (28.6) |
| Stem cell source, n (%) |  |
| Peripheral blood | 14 (22.2) |
| Cord blood | 11 (17.5) |
| Peripheral + cord blood ^#^ | 30 (47.6) |
| Not available ^*^ | 8 (12.7) |
| Conditioning intensity, n (%) |  |
| Myeloablative conditioning | 55 (87.3) |
| Not available ^*^ | 8 (12.7) |
| GVHD prophylaxis ^△^, n (%) |  |
| PTCy | 38 (60.3) |
| ATG | 34 (54.0) |
| GVHD, n (%) | 15 (23.8) |
| Post-transplant maintenance, n (%) | 4 (6.3) |
| Abbreviations: Allo-HSCT, allogeneic hematopoietic stem cell transplantation; CR, complete remission; PR, partial remission; MRD, minimal residual disease; PTCy, post-transplant cyclophosphamide; ATG, anti-thymocyte globulin; GVHD, graft-versus-host disease. ^#^, combined peripheral blood and umbilical cord blood transplantation. ^*^, data unavailable for patients transplanted at outside centers. ^△^, some patients received combined ATG and PTCy for GVHD prophylaxis. | |
